# Therapeutic efficacy of a novel self-assembled immunostimulatory siRNA combining apoptosis promotion with RIG-I activation in gliomas

**DOI:** 10.1186/s12967-024-05151-5

**Published:** 2024-04-29

**Authors:** Junxiao Chen, Ziyuan Liu, Haiting Fang, Qing Su, Yiqi Fan, Luyao Song, Shuai He

**Affiliations:** Department of Pharmacy, Zhujiang Hospital of Southern Medical University, Guangzhou, 510282 Guangdong China

**Keywords:** Immunostimulatory RNAs, RIG-I, Glioma, MHC-I, Apoptosis, IFN

## Abstract

**Background:**

Current cancer therapies often fall short in addressing the complexities of malignancies, underscoring the urgent need for innovative treatment strategies. RNA interference technology, which specifically suppresses gene expression, offers a promising new approach in the fight against tumors. Recent studies have identified a novel immunostimulatory small-interfering RNA (siRNA) with a unique sequence (sense strand, 5’-C; antisense strand, 3’-GGG) capable of activating the RIG-I/IRF3 signaling pathway. This activation induces the release of type I and III interferons, leading to an effective antiviral immune response. However, this class of immunostimulatory siRNA has not yet been explored in cancer therapy.

**Methods:**

Isi*BCL-2*, an innovative immunostimulatory siRNA designed to suppress the levels of B-cell lymphoma 2 (BCL-2), contains a distinctive motif (sense strand, 5’-C; antisense strand, 3’-GGG). Glioblastoma cells were subjected to 100 nM isi*BCL-2* treatment in vitro for 48 h. Morphological changes, cell viability (CCK-8 assay), proliferation (colony formation assay), migration/invasion (scratch test and Transwell assay), apoptosis rate, reactive oxygen species (ROS), and mitochondrial membrane potential (MMP) were evaluated. Western blotting and immunofluorescence analyses were performed to assess RIG-I and MHC-I molecule levels, and ELISA was utilized to measure the levels of cytokines (IFN-β and CXCL10). In vivo heterogeneous tumor models were established, and the anti-tumor effect of isi*BCL-2* was confirmed through intratumoral injection.

**Results:**

Isi*BCL-2* exhibited significant inhibitory effects on glioblastoma cell growth and induced apoptosis. *BCL-2* mRNA levels were significantly decreased by 67.52%. Isi*BCL-2* treatment resulted in an apoptotic rate of approximately 51.96%, accompanied by a 71.76% reduction in MMP and a 41.87% increase in ROS accumulation. Western blotting and immunofluorescence analyses demonstrated increased levels of RIG-I, MAVS, and MHC-I following isi*BCL-2* treatment. ELISA tests indicated a significant increase in IFN-β and CXCL10 levels. In vivo studies using nude mice confirmed that isi*BCL-2* effectively impeded the growth and progression of glioblastoma tumors.

**Conclusions:**

This study introduces an innovative method to induce innate signaling by incorporating an immunostimulatory sequence (sense strand, 5’-C; antisense strand, 3’-GGG) into siRNA, resulting in the formation of RNA dimers through Hoogsteen base-pairing. This activation triggers the RIG-I signaling pathway in tumor cells, causing further damage and inducing a potent immune response. This inventive design and application of immunostimulatory siRNA offer a novel perspective on tumor immunotherapy, holding significant implications for the field.

## Introduction

The occurrence of cancer is typically associated with abnormal mutations in tumor suppressor genes and oncogenes, such as the aberrant expression of the anti-apoptotic protein B-cell lymphoma 2 (BCL-2) [1]. Additionally, challenges in cancer treatment often arise from immune reconstitution and the immunosuppressive environment [2]. Cancer remains a leading cause of death, with approximately 28.4 million cases predicted by 2040 [3]. Worldwide, oncologists employ various conventional therapies for cancer treatment, including chemotherapy, radiotherapy, and surgery [4]. However, the high failure rate is attributed to drug resistance, drug toxicity, and severe side effects [5, 6]. Consequently, there is an urgent need for novel and effective cancer treatment methods.

In this context, nanotechnologies, represented by silver nanoparticles (AgNPs), and gene therapy technologies, represented by small-interfering RNA (siRNA), are expected to open new avenues for cancer treatment [7, 8]. Due to their distinctive physicochemical properties, including optical, thermal, and electrical conductivity, as well as their activity against bacteria, fungi, and viruses, AgNPs have demonstrated anti-tumor effects in in vitro and in vivo tumor models, rendering them suitable for certain cancer treatment strategies and tools [9–11]. Additionally, siRNA can hinder the proliferation and growth of tumor cells by suppressing highly expressed oncogenes in a tumor-specific manner. According to electronic databases such as Medline, Embase, Clinicaltrials.com, and Molecularmatch.com, and information from academic conferences hosted by the American Society of Clinical Oncology, the American Society of Hematology, the American Association for Cancer Research, and the European Society for Medical Oncology, 33 RNA-interference drugs were being tested in clinical trials for cancer treatment across all phases of clinical research, including active and terminated trials, as of February 2016 [8]. Notably, scientists have begun focusing on developing dual-functional siRNA capable of inducing immune stimulation to restore and reverse the tumor immunosuppressive environment. The combination of oncogene inhibition and immune system activation holds promise for achieving more effective anti-tumor immunotherapy.

Designing siRNA drugs to address the challenge of innate immune stimulation mediated by double-stranded RNA (dsRNA) has been a persistent endeavor, aiming to prevent adverse immune off-target effects and the misinterpretation of experimental results. As far back as two decades ago, studies identified that this immune off-target effect might involve intracellular RNA sensors, including protein kinase R, retinoic acid-inducible gene I (RIG-I), or toll-like receptors (TLRs), thereby stimulating innate antiviral immune responses. This stimulation can induce changes in the cellular transcriptome and proteome, hinder cell growth, and promote cell apoptosis. Currently, it is understood that factors contributing to these effects encompass siRNA structure, sequence, and delivery methods. For instance, unmodified siRNA has the potential to activate TLR7/8, and the presence of the “5’-UGUGU-3’” motif can activate TLR7. These effects can be regulated through chemical modifications or delivery systems [12–17]. While the activation of innate immunity by dsRNA is not universally desirable, it can prove beneficial in specific scenarios such as viral infections and cancer treatment [18]. In recent years, researchers have developed bifunctional 5’-ppp siRNAs capable of simultaneously activating RIG-I-mediated immune responses and inhibiting the expression of oncogenes or drug-resistant genes [19].

Recent research has identified a novel class of immunostimulatory RNAs characterized by a distinctive motif (sense strand, 5’-C; antisense strand, 3’-GGG). These RNAs activate the RIG-I/interferon regulatory factor 3 (IRF3) signaling pathway by forming RNA dimers through Hoogsteen base pairing, subsequently inducing the production of type I and type III interferons (IFNs) to induce antiviral responses [20]. The formation of RNA dimers in this particular class of siRNA leads to a length extension of approximately 50 base pairs. These dimerized RNA molecules lack overhangs and interact with RIG-I through the exposed 5’ terminus. Surprisingly, the precise length of the dimer allows for efficient activation of RIG-I, typically requiring an average length of 25 base pairs or longer for robust signal transduction, while minimizing excessive activation of other RNA recognition receptors such as TLR3 and melanoma differentiation-associated protein 5, which necessitate dsRNA exceeding 100 base pairs for optimal signaling [21]. This finely tuned mechanism helps maintain tissue integrity by preventing immunopathology resulting from a hyperactive immune response. Despite lacking bisphosphate or triphosphate moieties, a recent study has demonstrated RIG-I’s ability to recognize the 5’ end of longer dsRNA molecules [22]. Furthermore, these molecules can be synthesized using a simple and cost-effective process. While these immunostimulatory RNA-mediated immune responses have proven effective in inhibiting infection by various prevalent viruses, their feasibility in cancer immunotherapy remains to be established, as existing research findings supporting their efficacy are currently unavailable.

Gliomas, aggressive brain tumors with high BCL-2 levels, pose a significant therapeutic challenge due to their resistance to conventional treatments and high recurrence rates [23]. During glioma progression, tumor cells interact with their microenvironment, including immune cells, astrocytes, and endothelial cells, and secrete cytokines, chemokines, and other factors that facilitate immune evasion and support tumorigenesis [24, 25]. Therefore, an immunostimulatory siRNA containing a specific sequence (sense strand, 5’-C; antisense strand, 3’-GGG), referred to as isi*BCL-2*, was designed in this study, to target the anti-apoptotic protein BCL-2 for gene silencing. By being the first to incorporate this new immunostimulatory motif into cancer treatment, we have initiated an exploration of its potential as an anti-tumor agent and its immune-boosting properties.

## Materials and methods

### Cell culturing

The U251 human glioblastoma (GBM) cell line was procured from Procell Life Science & Technology (Wuhan, China). The cells were cultivated in Dulbecco’s modified Eagle medium (DMEM, Gibco, Billings, MT, USA) supplemented with 10% fetal bovine serum (FBS) and maintained in a humidified atmosphere with 5% CO_2_ at 37 °C.

### siRNA sequences

The siRNA sequences used in this study were as follows: si*Bcl-2* sense: 5′-GUACAUCCAUUAUAAGCUGdTdT-3′, si*Bcl-2* antisense: 5′-CAGCUUAUAAUGGAUGUACdTdT-3′; isi*BCL-2* sense: 5′-CCUGUACAUCCAUUAUAAGCUGUGG-3′, isi*BCL-2* antisense: 5′-CCACAGCUUAUAAUGGAUGUACAGGGG-3′; siNC sense: 5′-GUGAUUGCGAGACUCUGAdTdT-3′, siNC antisense: 5′-UCAGAGUCUCGCAAUCACGdTdT-3′; and siRIG-I sense: 5′-AGCACUUGUGGACGCUUUAAAdTdT-3′, siRIG-I antisense: 5′-UUUAAAGCGUCCACAAGUGCUdTdT-3′. All of these siRNAs were purchased from Suzhou GenePharma (Shanghai, China).

### Establishment of the in vitro assay

Cells were seeded into various types of plates (CORNING, Corning, NY, USA) at the recommended cell density per well and allowed to attach for 48 h. Cells in different groups underwent the following treatments. Cells in the control group received the same conditions as the other groups but without any treatment. In the siNC, si*BCL-2*, and isi*BCL-2* groups, the transfection reagent Lipofectamine 2000 (Invitrogen, Waltham, MA, USA) of a concentration of 100 nM was introduced into different wells for each group. The cells were then incubated in serum Opti-MEM for 4–6 h, followed by medium replacement with a medium containing 10% FBS for continued incubation for 48 h.

### Quantitative reverse transcription polymerase chain reaction (qRT-PCR)

To assess the mRNA levels of *Bcl-2*, *RIG-I*, *IFN-β*, and *C-X-C motif chemokine ligand 10* (*CXCL10*) in cancer cells, qRT-PCR was conducted. U251 cells were subjected to various drug treatments, and total RNA was extracted using a total RNA extraction kit (EZBiosciences, Roseville, MN, USA) following the manufacturer’s protocols. Reverse transcription of RNA into cDNA was performed using a Color Reverse Transcription Kit (EZBiosciences). qRT-PCR was performed using SYBR Green Color qPCR Mix (EZBiosciences) following the manufacturer’s instructions and analyzed using an ABI fluorescence quantitative PCR instrument (QuantStudio 3&5; Thermo Fisher, Waltham, MA, USA). The primers used for PCR amplification were as follows. *Bcl-2* sense: 5′-GACTTCTCCCGCCGCTACCG-3′, *Bcl-2* antisense: 5’-ACACACACATGACCCCACCGAAC-3’; *RIG-I* sense: 5′-AGGCAGAGGAAGAGCAAGAGGTAG-3′, *RIG-I* antisense: 5’-CTTTGGCTTGGGATGTGGTCTACTC-3′; *IFN-β* sense: 5′-CTTGGATTCCTACAAAGAAGC-3′, *IFN-β* antisense: 5′-CATCTCATAGATGGTCAATGC-3′; and *CXCL10* sense: 5’- CTTCCAAGGATGGACCACACA-3′, *CXCL10* antisense: 5′-CCTTCCTACAGGAGTAGTAGCAG-3′. The relative expression of the target genes was normalized to the *GAPDH* control using the 2^−ΔΔCt^ method.

### Cell viability assay

The determination of cell viability was conducted through the cell counting kit 8 (CCK-8) assay (Fude, Hangzhou, China). Briefly, cancer cells were initially seeded in 96-well plates at a density of 3 × 10^3^ cells per well. Following 24 h incubation and 48 h treatment with various drugs, the CCK-8 reagent was introduced into each well and incubated for 1–4 h as per the provided instructions. Subsequently, the optical density values were measured at 490 nm using an enzyme-linked immunosorbent assay (ELISA) reader.

### Colony formation ability

U251 cells were seeded in six-well plates at a density of 2 × 10^3^ cells per well. Following a 24-hour incubation period, the cells were subjected to treatment either without or with a specified amount of drug for 14 days. Subsequently, the cells were fixed with 4% paraformaldehyde for 10 min and stained with 0.05% crystal violet for 30 min. The number of individual colonies containing at least 50 cells was manually counted under a microscope.

### Assessment of cancer cell migration and invasion

For the migration assay, U251 cells were plated in six-well plates at a density of 2 × 10^5^ cells per well. Once the cell growth reached 100% confluence, a sterile 200 µL pipette tip was used to directly create a scratch in the cell layer. The cells were then treated with or without a specified amount of drug, and the distance traveled by the cells between the two boundaries of the scratched area was recorded at 0, 12, 24, 36, and 48 h using phase-contrast microscopy.

For the invasion assay, U251 cells (1 × 10^4^) were placed in the upper chamber of a 24-well Transwell chamber with a polycarbonate membrane (8 μm pore size; Corning). After 4-hour incubation, the cells were treated with or without a specified amount of drug in a serum-free DMEM medium. Following an additional 48 h incubation, the cells from the upper well were gently removed with a cotton swab. Cells that migrated through the filter membrane to the bottom chamber were washed with phosphate-buffered saline (PBS), fixed with methanol for 30 min at 25 °C, and stained with a 0.25% crystal violet solution. The quantification of migrated cells was conducted by counting five randomly selected fields of view per filter under an inverted microscope. For the invasion assay, 100 µL diluted matrix gel was added vertically to the center of the Transwell chamber and incubated at 37 °C for 4–5 h. Subsequently, cancer cells (1.5 × 10^4^) were inoculated into the upper chamber, and the remaining procedures were performed as described in the migration assay.

### Determination of apoptosis

To assess isi*BCL-2*-induced apoptosis in cancer cells, Annexin V/propidium iodide (PI) double staining was conducted. U251 cells were seeded at a density of 2 × 10^5^ cells per well in a six-well plate and subjected to treatment with or without a specific amount of drug. After 48-hour treatment, the cellular status was observed through electron microscopy. Subsequently, cells were harvested and stained with Annexin V/PI using the Annexin V/PI Double Stain Apoptosis Detection Kit (Elabscience, Wuhan, China) following the manufacturer’s protocols. The stained cells were then analyzed using a CytoFLEX flow cytometer (Beckman Coulter, Brea, CA, USA).

### Western **blotting analysis**

In this study, the following antibodies were employed: BCL-2 (T40056T40056; Abmart, Shanghai, China), BAX (T40051; Abmart), Cytochrome C (T55734T55734; Abmart), PARP (T40050; Abmart), Caspase 3 (T40044; Abmart), Caspase 9 (T40046; Abmart), RIG-I (#20566-1-AP; Proteintech, Wuhan, China), mitochondrial antiviral signaling protein (MAVS, #66911-1-Ig; Proteintech), IRF3 (#11312-1-AP; Proteintech), P-IRF3 (#29528-1-AP; Proteintech), and GAPDH (#60004-1-Ig; Proteintech). Cells were harvested and lysed in radio-immunoprecipitation assay buffer (BL504A; Biosharp, Tallinn, Estonia) supplemented with Halt protease (BS-00-0903; Biosharp) and phosphatase inhibitor cocktail (BL615A; Biosharp) on ice. Subsequently, the cell lysates underwent Western blotting analysis, with GAPDH serving as a loading control.

### ELISA

Cytokines in mouse tumor tissue, as well as human IL-6 and IFN-β in cell culture medium, were detected using the Human IFN-β and CXCL10 ELISA Kit (Jiangsu Meiman, China) following the manufacturer’s instructions.

### Confocal immunofluorescence microscopy

Following rinsing with PBS, cells were fixed with 4% paraformaldehyde for 30 min, rapidly blocked using QuickBlock™ Blocking Buffer (P0220; Beyotime, Shanghai, China) for 15–20 min, and then incubated with the primary antibody diluted in blocking buffer (1% goat serum in PBS-Tween). The antibodies were subjected to overnight incubation at 4 °C. Subsequently, cells were incubated with a fluorescent secondary antibody, CoraLite488/594 (Proteintech), for 1 h at 25 °C. After staining with the secondary antibody, nuclei were counterstained with DAPI (BS097-10 mg; Biosharp). Fluorescence imaging was carried out using a confocal laser scanning microscope (AX NIS-Elements v5.4; Nikon, Tokyo, Japan), and image processing was conducted using NIS-Elements software (Nikon).

### Animal handling

BALB/c nude mice (female, 4–6 weeks old, weighing approximately 20 g) were obtained from Sun Yat-sen University Laboratory Animal Center (Guangzhou, China) and housed in a sterile environment in accordance with standardized animal care protocols. The experiments were conducted in compliance with national regulations.

### Assessment of isi*BCL-2* anti-tumor activity in vivo

Human glioma U251 cells (1 × 10^7^) were subcutaneously injected into the right thigh of female nude BALB/c mice (4–6 weeks old, weighing 18–20 g). The mice were randomly divided into three groups, each consisting of five mice: control (PBS solution), si*BCL-2* (2.5 nmol/20 g), and isi*BCL-2* (2.5 nmol/20 g). Intratumoral injections were administered when the tumors reached approximately 80 mm^3^. PBS, si*Bcl-2*, and isi*Bcl-2* were used with the in vivo transfection reagent (Entranster^TM^-in vivo; Engreen Biosystem, Beijing, China) every three days for 22 days. Subsequently, tumor samples and blood samples were collected from the mice. Tumor size was measured every two days using vernier calipers, and tumor volume was calculated using the formula: tumor volume: Tumor volume (mm^3^) = 0.5 × length × width^2^. Tumor samples were processed by cutting to a specific weight and homogenized for qRT-PCR and Western blot analysis. Blood samples were centrifuged, and the upper layer of serum was collected and diluted for ELISA analysis.

### Immunohistochemistry (IHC) and terminal deoxynucleotidyl transferase dUTP nick end labeling (TUNEL) assay

Mice were subjected to treatment with PBS, si*Bcl-2*, and isi*Bcl-2*. After a 22-day treatment period, the mice were euthanized, and tumor tissues were excised. The excised tumor tissues were fixed with 4% paraformaldehyde, embedded in paraffin, sliced into 4–8 μm sections, and subjected to IHC staining. Anti-Ki67 (GB121141; Servicebio, Wuhan, China), anti-Bcl-2, anti-RIG-I, and anti-major histocompatibility complex class I (MHC-I) antibodies were applied for incubation at 4 °C for 24 h. Subsequently, tissue sections were incubated with biotin-labeled secondary antibodies for 1 h and stained with 3,3’-diaminobenzidine substrate. After hematoxylin restaining and dehydration, the tissue sections were sealed with coverslips, and images were scanned using a high-capacity digital slide scanner system (3DHISTECH, Budapest, Hungary).

For the TUNEL assay, the dewaxed and hydrated tissue sections underwent incubation with proteinase K solution for 30 min at 37 °C, followed by three washes with PBS. Each section was then incubated with 50 µL TUNEL reaction solution, protected from light, at 37 °C for 2 h. Subsequently, the sections were washed three times with PBS, incubated with DAPI staining solution for 10 min at 37 °C, washed with PBS, and dried. Finally, the sections were sealed with anti-fluorescence quenching sealer, and images were captured using a Nikon confocal microscope (AX NIS-Elements v5.4).

### Statistical analysis

Statistical analyses were performed using SPSS v17.0 (SPSS, Chicago, IL, USA). The data are presented as the mean ± standard deviation (SD) of at least three independent experiments. Differences between groups were assessed using Student’s *t*-tests or two-way analysis of variance, with the significance level established at *p < 0.05.

## Results

### Isi*BCL-2* significantly inhibits the growth of glioma cells and suppresses migration and invasion

**Fig. 1:**
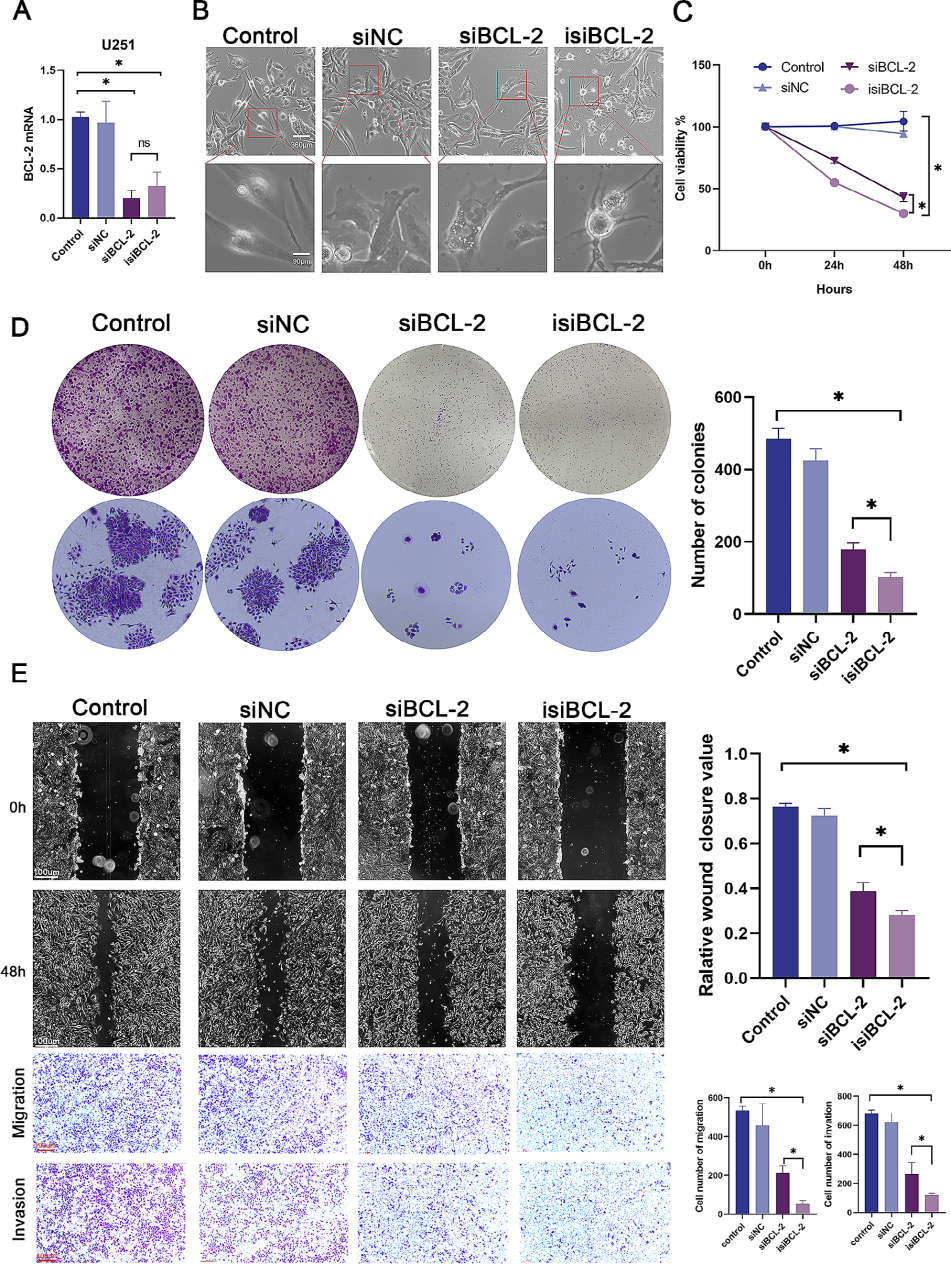
Isi*BCL-2* significantly inhibits the growth of glioma cells. **A**. Relative *BCL-2* mRNA levels were quantified by qRT-PCR in U251 cells 48 h after transfection with siNC, si*BCL-2*, or isi*BCL-2* (100 nM). **B**. Morphological analysis of U251 cells 48 h after transfection with indicated RNAs. Scale bars: 360 μm and 90 μm. **C–D**. CCK-8 assay and colony formation assay of U251 cells. **E**. Scratch wound healing assay and Transwell analysis performed to further confirm the effect of isi*BCL-2* on migration and invasion in U251 cells. Scale bar: 100 μm. Data are presented as mean ± standard deviation (*n* = 3), *p < 0.05 vs. control

To evaluate the knockdown efficiency of the newly synthesized isi*BCL-2* molecule, a transfection experiment was conducted in glioma cells, followed by the quantification of *BCL-2* mRNA levels using RT-qPCR. The results, depicted in Fig. 1A, conclusively demonstrate that transfection with isi*BCL-2* leads to significant suppression of *BCL-2* levels in U251 cells. Additionally, microscopic examination reveals distinct apoptotic characteristics such as cellular crumpling, rounding, and detachment in cell lines following drug transfection (Fig. 1B). Furthermore, the CCK-8 assay indicates a comparable inhibition of cell viability in the glioma cell lines (Fig. 1C). To further confirm the inhibitory effects of isi*BCL-2* on cancer cell growth, a colony formation assay was conducted, demonstrating a strong suppression of colony formation in cells transfected with isi*BCL-2* (Fig. 1D). These findings establish the significant inhibitory effect of isi*BCL-2* on cancer cell growth.

As BCL-2 functions not only in apoptosis but also in cancer cell metastasis as an oncogene, the impact of the drug on cancer cell migration and invasion was investigated [1]. The monolayer scratch healing assay indicated a significant inhibition of migration in U251 cells transfected with isi*BCL-2*, as illustrated in Fig. 1E. Additionally, Transwell assays provided further evidence of the inhibitory effects of isi*BCL-2* on both cancer cell migration and invasion (Fig. 1E).

### Isi*BCL-2* induces apoptosis and activates the RIG-I signaling pathway in glioma cells

**Fig. 2:**
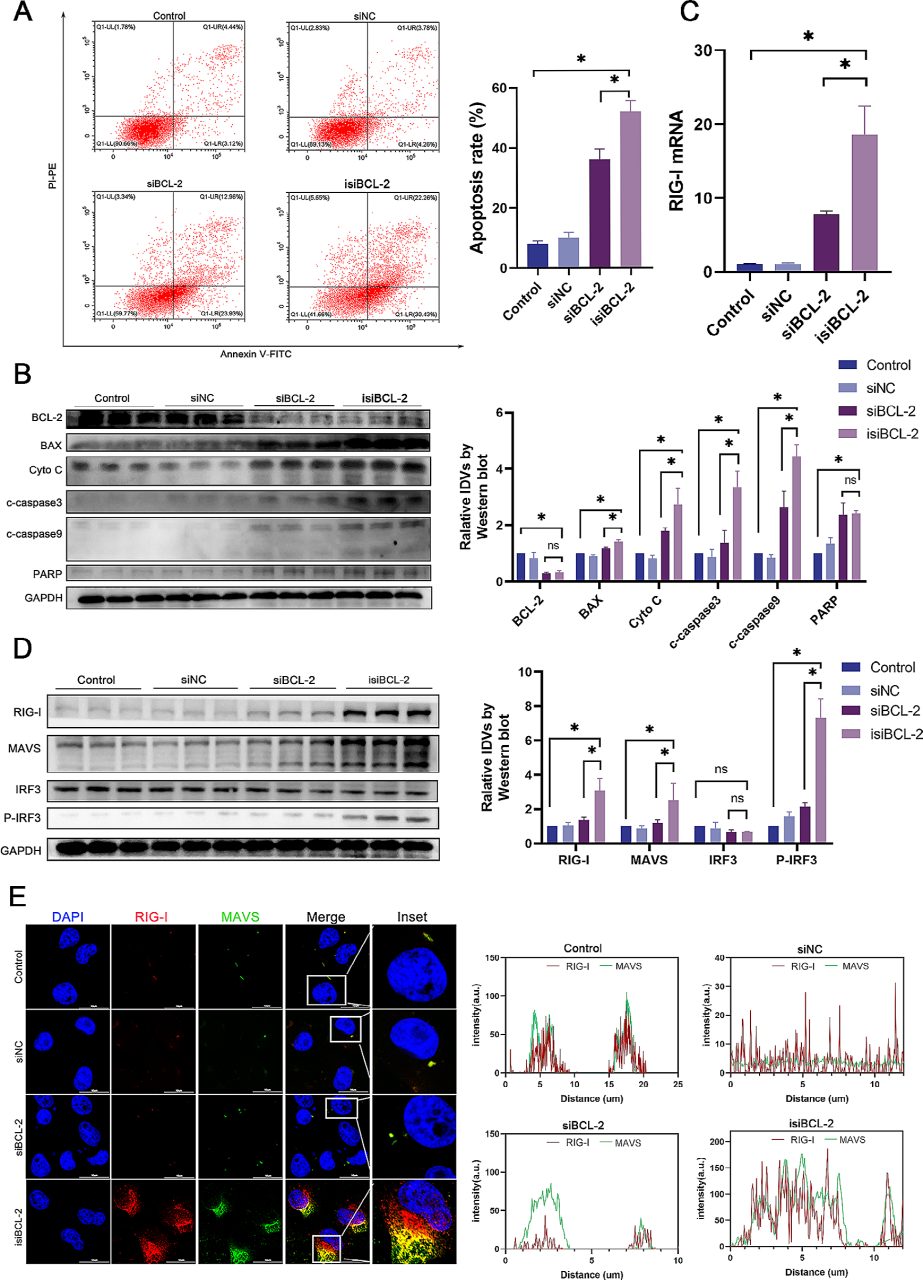
Isi*BCL-2* induces apoptosis and activates the RIG-I signaling pathway in glioma cells. **A**. Flow cytometry analysis of apoptosis in U251 cells 48 h after transfection with indicated RNAs. **B**. Western blotting analysis of protein levels of BCL-2, BAX, cytochrome C, cleaved caspase-3, cleaved caspase-9, and PARP in U251 cells 48 h after transfection with indicated RNAs. **C**. Quantitative analysis of *RIG-I* mRNA levels using qRT-PCR after a 48-h treatment with indicated RNAs. **D**. Western blotting analysis of protein levels of RIG-I, MAVS, IRF3, and P-IRF3 in U87 and U251 cells 48 h after transfection with indicated RNAs. **E**. Confocal fluorescence microscopy images of RIG-I and MAVS levels. Scale bar: 50 μm. Data are presented as mean ± standard deviation (*n* = 3). *p < 0.05 vs. control

To explore the apoptotic potential of isi*BCL-2* in cancer cells, Annexin V- fluorescein isothiocyanate and PI double-staining were utilized. As depicted in Fig. 2A, there was a significant increase in the number of apoptotic cells in U251 cells transfected with isi*BCL-2* compared to that in the control group. Notably, isi*BCL-2* demonstrated a greater capacity to induce apoptosis than si*BCL-2*. Furthermore, the impact of isi*BCL-2* on the levels of apoptosis-related proteins was verified using Western blotting analysis. The results revealed the downregulation of BCL-2 protein expression and a significant upregulation of the expression of pro-apoptotic proteins such as Bax, Cyto C, PARP, c-caspase3, and c-caspase9 in U251 cells following transfection with isi*BCL-2* (Fig. 2B). These findings collectively suggest that isi*BCL-2* induces apoptosis in tumor cells by regulating apoptosis-related proteins.

To investigate the potential activation of the RIG-I signaling pathway by isi*BCL-2*, RT-qPCR experiments were conducted to assess the mRNA level of *RIG-I* (*DDX58*). As presented in Fig. 2C, U251 cells exhibited an upregulation expression of *RIG-I* upon transfection with isi*BCL-2* compared to that in the control group and si*BCL-2*. Immunofluorescence (Fig. 2D) and Western blotting (Fig. 2E) analyses further confirmed the upregulation of *RIG-I* levels, as well as its adaptor MAVS, and increased phosphorylation of IRF3 [26]. Phosphorylated IRF3 is pivotal for transcriptional activity and subsequent translocation into the nucleus as a transcription factor that induces IFN-β secretion [27, 28].

### Activation of the RIG-I signaling pathway induces dysregulation of mitochondrial membrane potential (MMP) and production of reactive oxygen species (ROS) in glioma cells

**Fig. 3:**
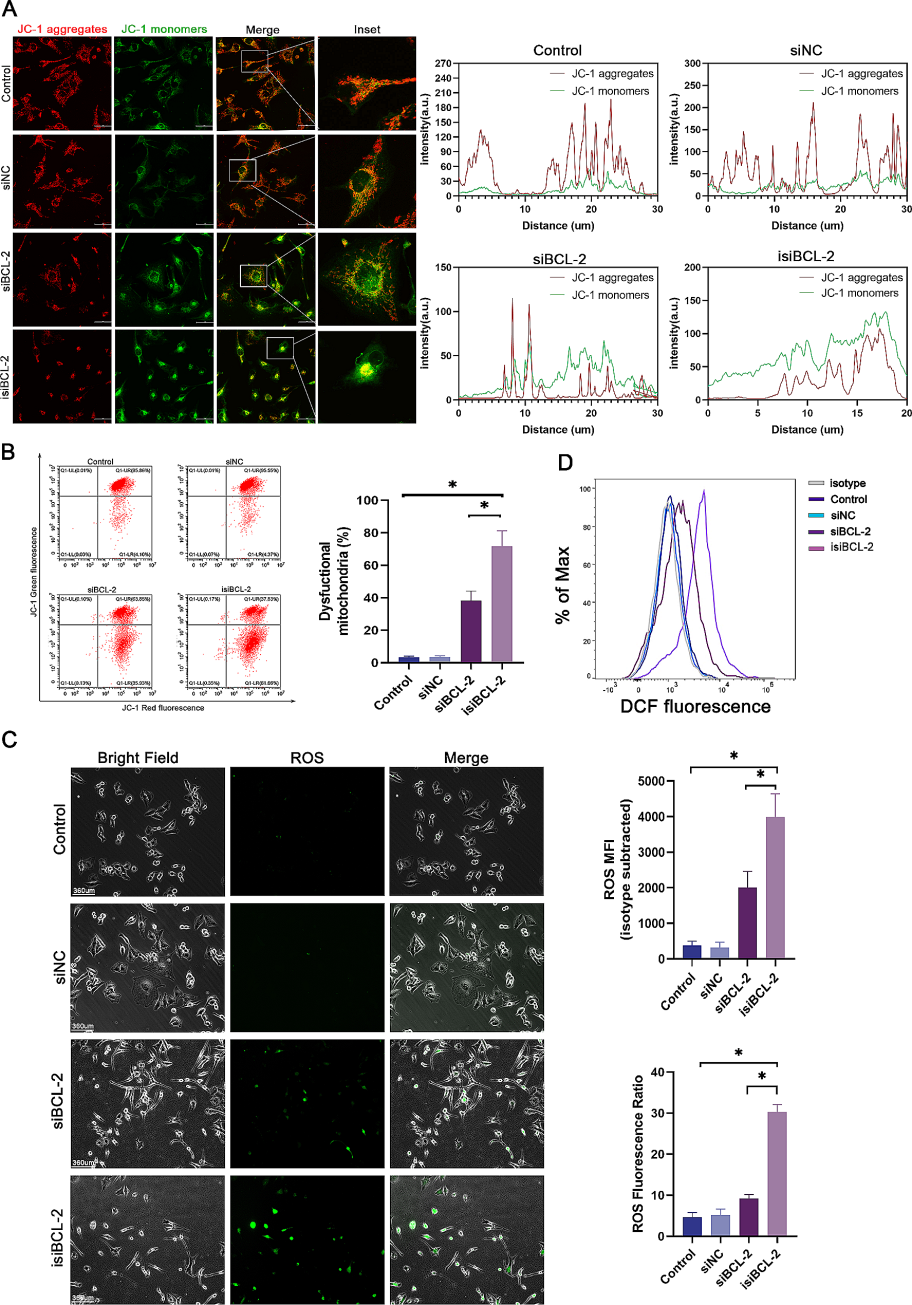
Isi*BCL-2* induces dysregulation of mitochondrial membrane potential (MMP) in glioma cells. **A–B**. Measurement of MMP using JC-1 staining in U251 cells transfected with indicated RNAs for 48 h, assessed by confocal fluorescence microscopy and flow cytometry. Scale bar: 50 μm. **C–D**. Assessment of ROS generation using DCFH-DA with fluorescence microscopy and flow cytometry. Scale bar: 360 μm. Data are presented as mean ± standard deviation (*n* = 3). *p < 0.05 vs. control

The activation of the RIG-I signaling pathway triggers the innate immune response and promotes ROS production as a defense mechanism against viral infections [29]. However, cancer cells often possess an elevated antioxidant capacity, acting as a barrier to pharmacological ROS damage. Consequently, novel strategies are needed to induce ROS generation in cancer cells [30]. Previous studies have indicated that RIG-I activation disrupts autophagic flux, leading to the accumulation of dysfunctional mitochondria, and ultimately generating detrimental ROS that contribute to cancer cell death [31]. Additionally, MAVS-mediated antiviral signaling has been associated with increased ROS production, altered mitochondrial morphology, mitochondrial dysfunction, and a significant loss of MMP (designated ΔΨm) [32, 33]. These experimental results demonstrate that isi*BCL-2* significantly reduces MMP in U251 cells (Fig. 3A–B), accompanied by the accumulation of ROS (Fig. 3C–D), thereby causing additional damage to cancer cells.

### Isi*BCL-2* stimulates the levels of MHC-I molecules and promotes cytokine release

The activation of the RIG-I receptor is widely acknowledged as a potent trigger for robust antiviral immune responses, characterized by the induction of type I IFNs (e.g., IFN-α and IFN-β). These cytokines play a pivotal role in mediating the intricate interaction between tumors and the immune system [34]. Furthermore, RIG-I activation leads to the upregulation of various innate immune response genes, including *CXCL10* [35], a potent chemokine responsible for immune cell recruitment to inflammatory sites. Additionally, stimulation of the RIG-I pathway results in increased levels of MHC-I molecules. This upregulation facilitates the efficient presentation of tumor-specific antigens, enabling their recognition and subsequent elimination by the immune system, primarily through the activation of dendritic cells and cytotoxic T cells [36–39].

**Fig. 4:**
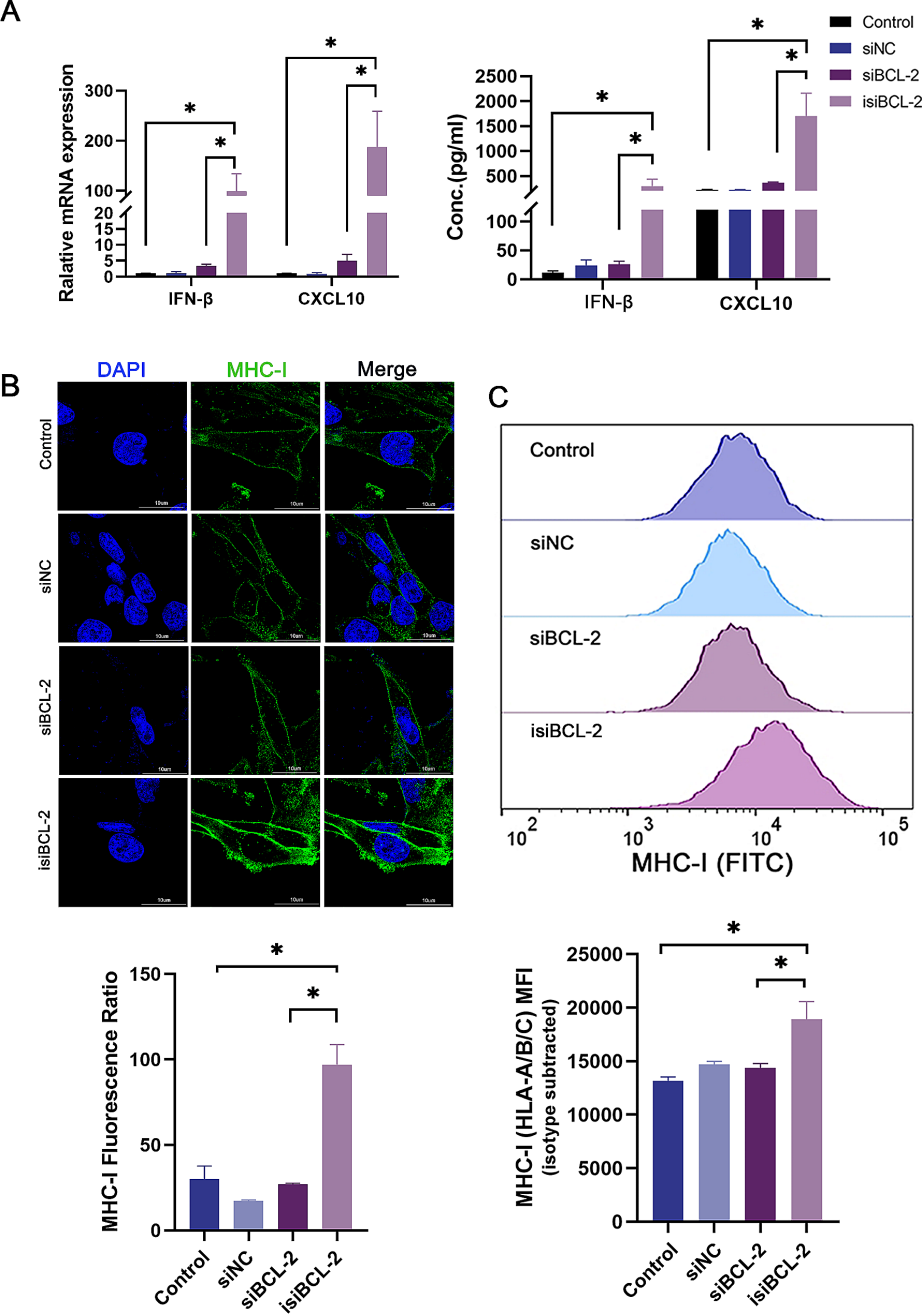
isi*BCL-2* induces MHC-I and cytokine levels in glioma cells. **A**. Detection of *IFN-β* and *CXCL10* levels using qRT-PCR and ELISA kits. **B–C**. Immunofluorescence micrographs and flow cytometry illustrating the levels of MHC-I in U251 cells transfected with indicated RNAs at 48 h post-transfection. Scale bar: 10 μm. Data are presented as mean ± standard deviation (*n* = 3). *p < 0.05 vs. control

The experimental findings presented herein provide compelling evidence that transfection with isi*BCL-2* induces a significant upregulation of mRNA expression of *IFN-β* and *CXCL10* in U251 cells (Fig. 4A). Moreover, the secretion of IFN-β and CXCL10 is significantly increased following isi*BCL-2* transfection, as confirmed by robust ELISA results (Fig. 4A). Immunofluorescence and flow cytometry analyses further corroborate these observations, revealing a substantial enhancement in MHC-I levels in U251 cells upon isi*BCL-2* transfection compared to those in both control and si*BCL-2*-treated cells (Fig. 4B–C). Collectively, these findings firmly establish the ability of the synthesized isi*BCL-2* to effectively induce the upregulation of type I IFNs, chemokines, and MHC-I levels.

### The effect of isi*BCL-2* on cell viability, apoptosis, cytokine release, and MHC-I upregulation is associated with RIG-I activation

**Fig. 5:**
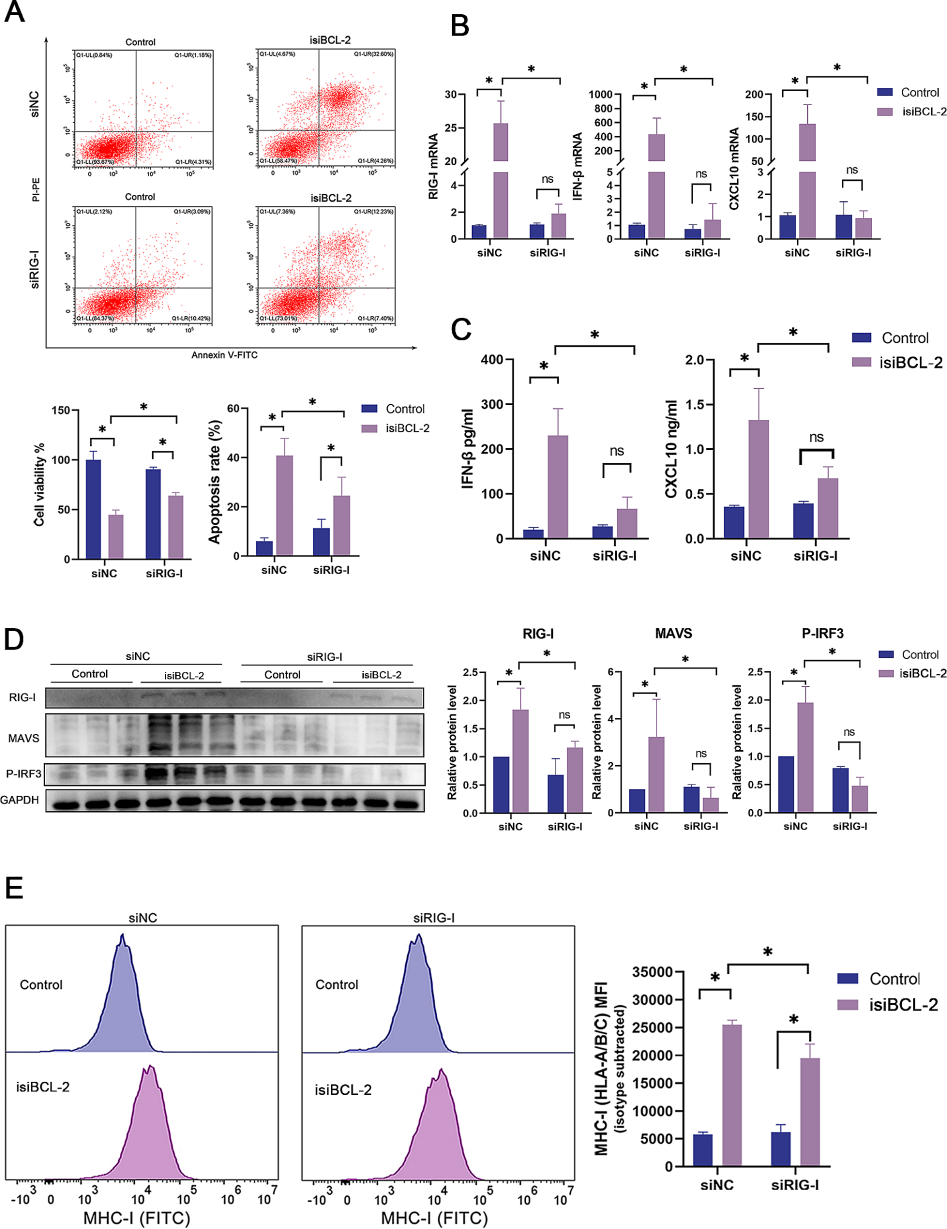
Effects of isi*BCL-2* on cell viability, apoptosis, cytokine release, and upregulation of MHC-I require RIG-I activation. **A**. Assessment of cell viability and apoptosis in U251 cells using CCK-8 and flow cytometry assays. **B**. Quantification of *RIG-I*, *IFN-β*, and *CXCL10* mRNA levels by qRT-PCR. **C**. Quantitative analysis of IFN-β and CXCL10 secretion levels using ELISA. **D**. Western blotting analysis of protein levels of RIG-I, MAVS, and P-IRF3. **E**. Flow cytometry analysis of MHC-I levels. Data are presented as mean ± standard deviation (*n* = 3). *p < 0.05 vs. control

Previous studies have suggested that the activation of the RIG-I signaling pathway induces intrinsic apoptosis in tumor cells, with tumor cells exhibiting higher susceptibility than non-malignant cells [31, 40]. CCK-8 assay and flow cytometry analysis revealed that the knockdown of *RIG-I* diminished the ability of isi*BCL-2* to impair cancer cell viability and induce apoptosis (Fig. 5A).

To investigate the impact of the RIG-I signaling pathway on cytokine release and MHC-I upregulation in isi*BCL-2*-induced cancer cells, U251 cells were pretreated with siRNA-RIG-I, and the mRNA levels of *IFN-β*, *CXCL10*, and *MHC-I* were assessed following isi*BCL-2* transfection. The results demonstrated decreased secretion of IFN-β and CXCL10 (Fig. 5B–C), as well as a reduction in MHC-I levels (Fig. 5E). Additionally, a decrease in MAVS protein levels and IRF3 phosphorylation was observed (Fig. 5D).

### Isi*BCL-2* induces apoptosis and upregulates MHC-I and cytokines in tumor tissues in vivo

**Fig. 6:**
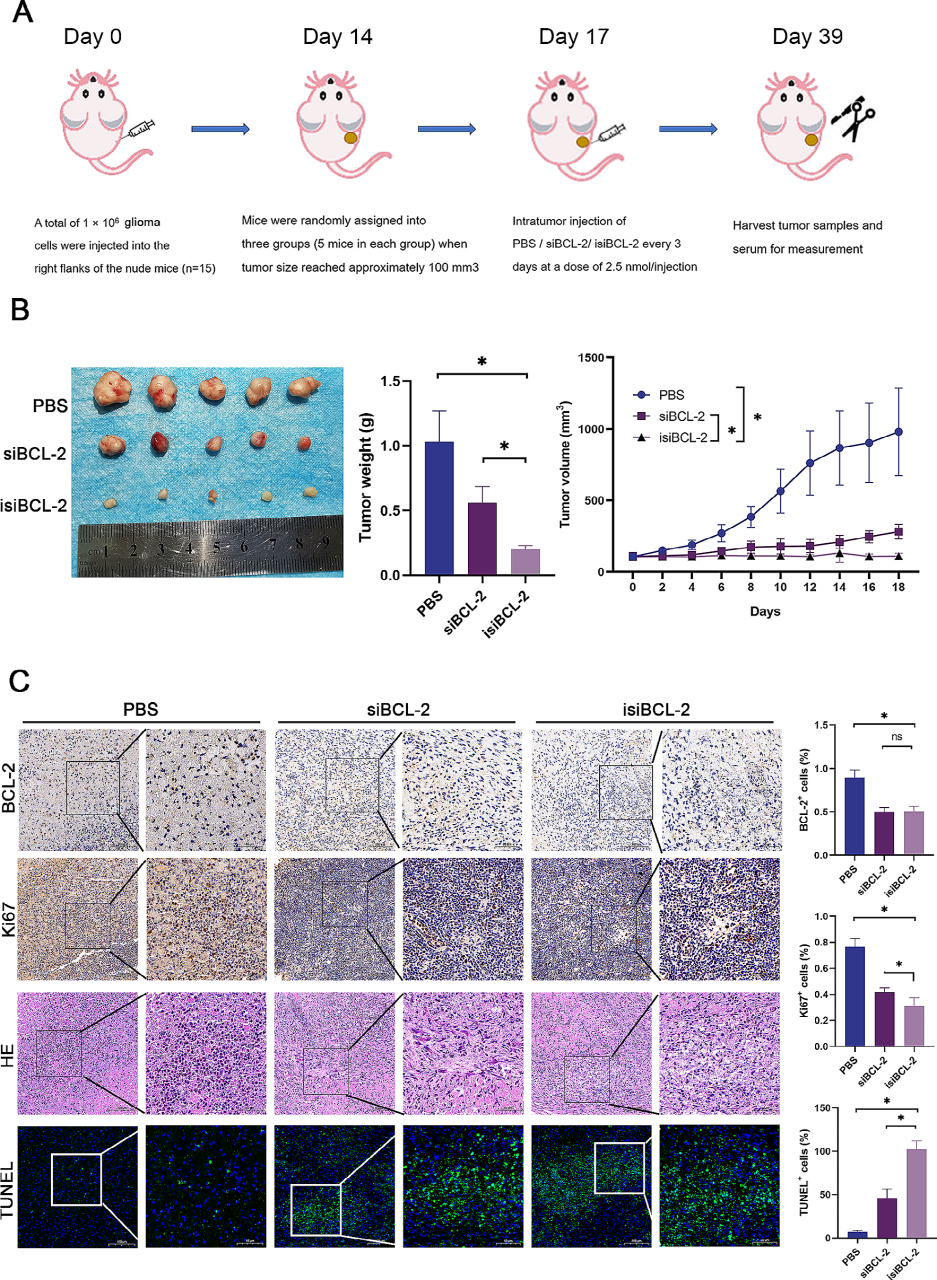
isi*BCL-2* Exerts antitumor effects in vivo. **A**. Establishment of a xenograft nude mice model bearing human glioma U251 cells, with subsequent treatment using a specified amount of isi*BCL-2*. **B**. Removal, photography, and weighing of tumors after 22 days of RNA treatment. Tumor volume and mouse body weight were monitored every two days. **C**. Immunohistochemical (IHC) analysis, hematoxylin and eosin (H&E) staining, and TUNEL assay of tumors excised from U251 tumor-bearing mice. BCL-2 and Ki67 protein levels in the tumor tissue assessed using IHC staining. The apoptosis rate in each group was determined using H&E staining and TUNEL assay. Scale bar: 40 μm Data are presented as mean ± standard deviation (*n* = 3). *p < 0.05 vs. PBS (control)

Subsequently, the in vivo anti-tumor efficacy of isi*BCL-2* was assessed using a Balb/C mouse model with subcutaneously implanted glioma cells on the right side (Fig. 6A). Intratumoral injection of both si*BCL-2* and isi*BCL-2* significantly inhibited glioma cell growth, with the isi*BCL-2* group exhibiting a more pronounced inhibitory effect (Fig. 6B). Immunohistochemical staining, hematoxylin and eosin staining, and TUNEL staining were conducted to examine proliferation activity and apoptosis in tumor tissues. The results revealed a significant reduction in BCL-2 and Ki67 levels, indicating suppressed tumor proliferation activity and the induction of apoptosis by isi*BCL-2 in vivo* (Fig. 6C).

**Fig. 7:**
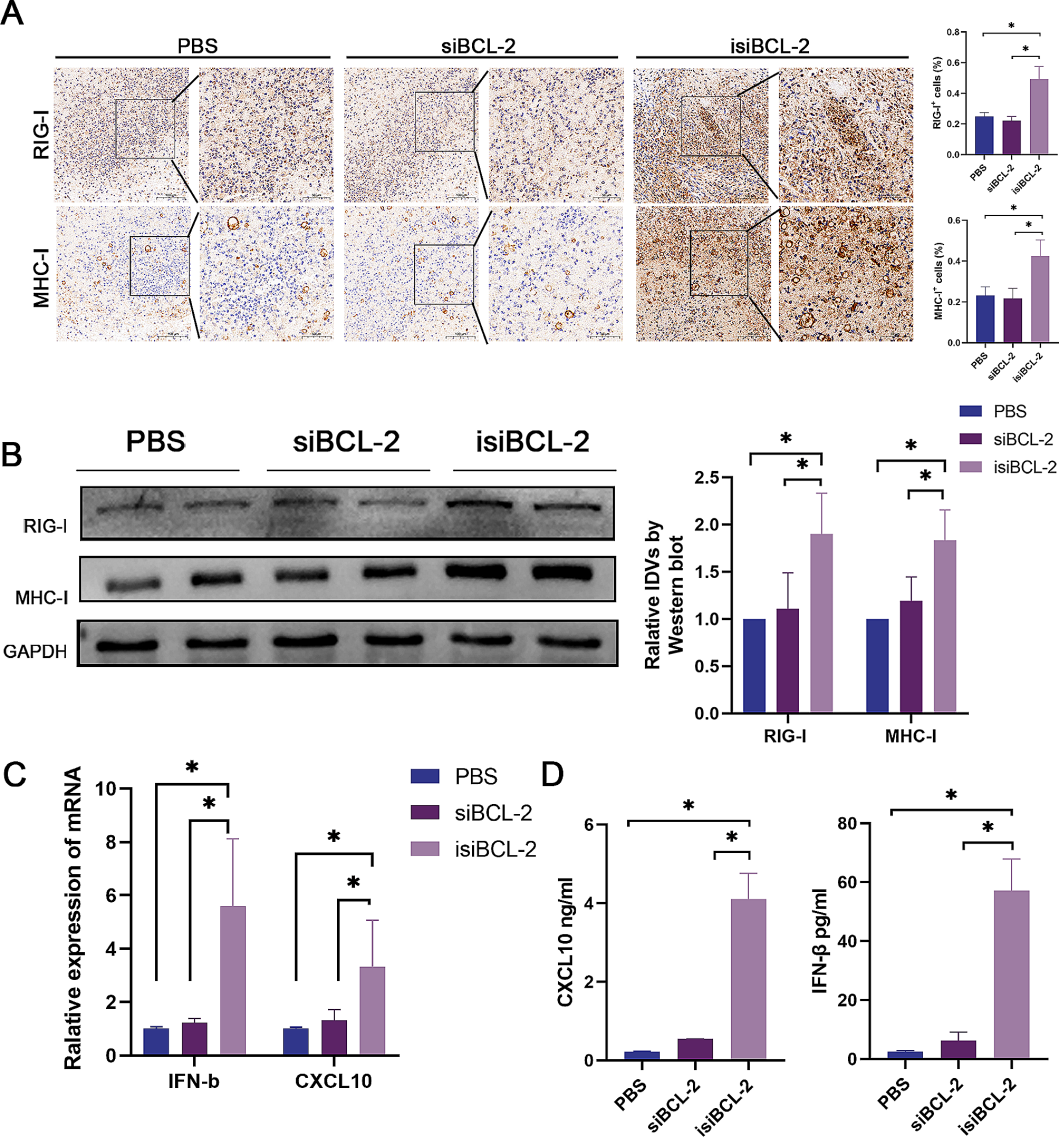
Analyses of RIG-I activation and levels of MHC-I and cytokine in tumor tissues. **A–B**. Assessment of RIG-I and MHC-I protein levels in tumor tissue using IHC staining and Western blotting analyses. Scale bar: 50 μm and 20 μm. **C–D**. Measurement of *IFN-β* and *CXCL10* levels in tumor tissue using qRT-PCR and ELISA. Data are presented as mean ± standard deviation. *n* ≥ 3, *p < 0.05 vs. PBS (control)

Furthermore, immunohistochemical staining and Western blotting analysis were performed to evaluate the levels of immunologically relevant proteins (e.g., RIG-I and MHC-I) in tumor samples. RT-qPCR and ELISA analyses were performed to measure the levels of IFN-β and CXCL10 in tumor tissues. The results demonstrated an upregulation of both RIG-I and MHC-I in tumor tissues following intratumoral injection of isi*BCL-2* (Fig. 7A–B), along with elevated levels of IFN-β and CXCL10 (Fig. 7C–D).

**Fig. 8:**
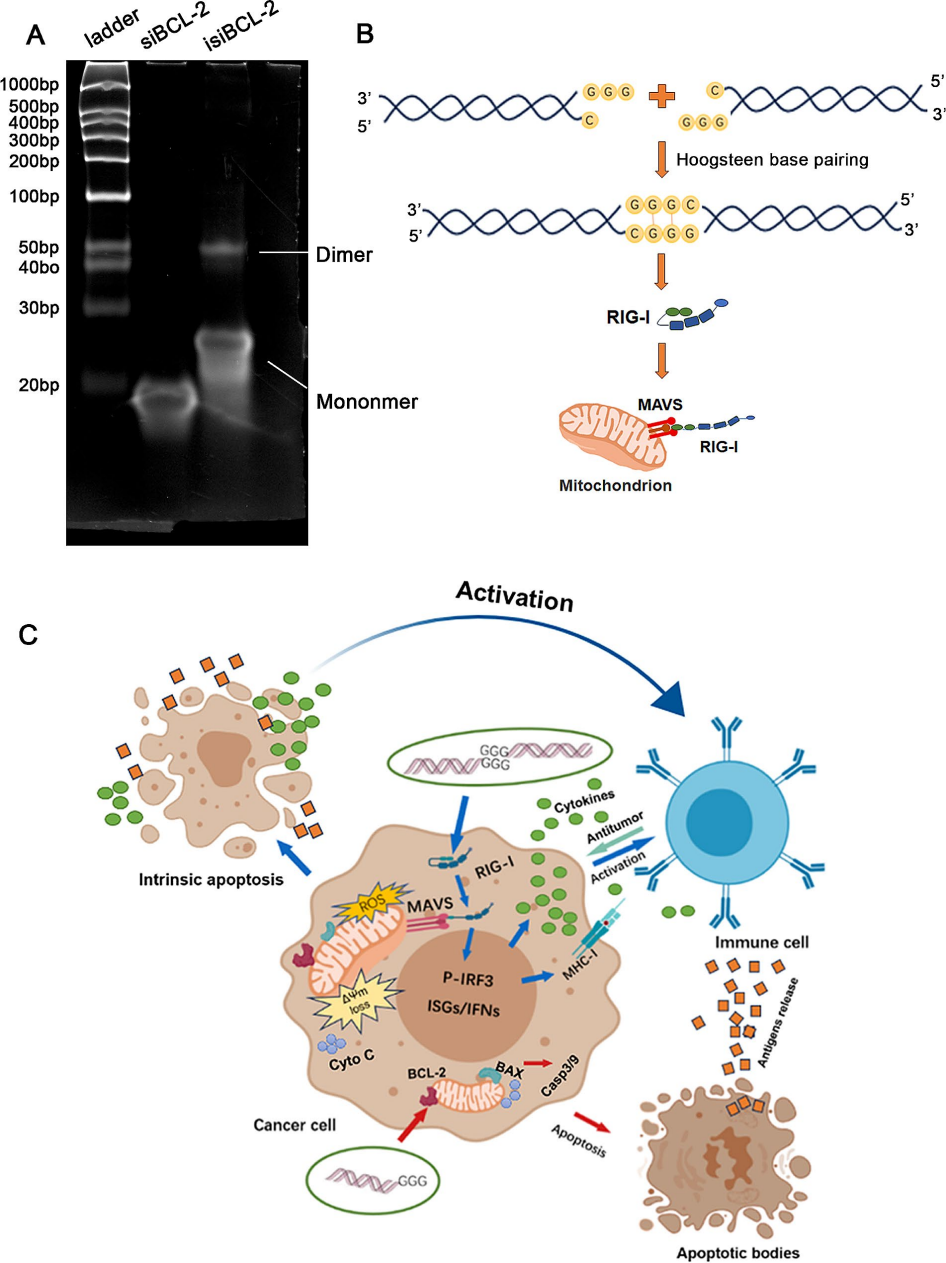
Mechanism of RNA dimer formation and tumor cell killing by isi*BCL-2* molecules. **A**. Electrophoretic image illustrating isi*BCL-2* molecules forming RNA dimers. **B**. Mechanism elucidating the formation of RNA dimers by isi*BCL-2* molecules. **C**. Mechanism elucidating immune activation and tumor cell eradication by isi*BCL-2*.

Finally, gel electrophoresis experiments were conducted to investigate the ability of the isi*BCL-2* molecule to form RNA dimers through Hoogsteen base pairing. The experiment results showed that the electrophoretic lane containing isi*BCL-2* produced bands at a molecular weight of ~ 50 bp (Fig. 8A). In contrast, the normal si*BCL-2* molecule did not produce these bands. This provides compelling evidence that the newly synthesized isi*BCL-2* molecule possesses the capability to form RNA dimers.

## Discussion

During tumorigenesis, dysregulation of apoptosis mechanisms often results from the upregulation of the anti-apoptotic protein BCL-2, leading to the malignant proliferation of tumor cells [1]. Furthermore, immune reconstruction and the tumor’s immune-suppressive microenvironment significantly reduce treatment efficacy, posing further complexity to the situation [2]. The objective of this study is to develop a dual-function siRNA capable of effectively silencing oncogenes while inducing appropriate immune stimulation. By inhibiting tumor growth and promoting apoptosis, this siRNA can also restore and reverse the tumor’s immune environment, transforming a “cold” tumor into a “hot” tumor, thereby enhancing the effectiveness of anti-tumor immune therapy.

SiRNA, a common tool and drug for gene silencing in biomedical research, has rapidly advanced over the past two decades [41]. Although the immunogenicity of siRNA is not widely accepted in practical use, it may offer potential benefits in certain treatment methods. In recent years, some dual-functional siRNAs have been designed to target TLRs, such as D-siRNA with uridine protrusion modifications targeting TLR8 activation and CpG-siRNA targeting TLR9 activation [42, 43]. Moreover, there is a growing preference for dual-functional siRNAs designed to activate RIG-I, such as 5’-ppp siRNAs designed for cancer therapy. These siRNAs suppress cancer genes or drug-resistant genes while activating RIG-I-mediated immune responses, providing therapeutic advantages in treating viral infections and various diseases, including cancer [18, 19]. Combining gene knockout with RIG-I activation represents a promising approach for anticancer therapy. Two previous studies successfully developed bifunctional siRNAs that effectively targeted and inhibited BCL-2 and TGF-β while simultaneously activating RIG-I through modification of the triphosphate group at the 5’ end of the siRNA, implementing these modifications to potentiate the anti-tumor effects of the siRNAs [35, 36].

A recent study has identified a novel RNA sequence motif (sense strand, 5’-C; antisense strand, 3’-GGG) that activates the RIG-I/IRF3 pathway, promoting the secretion of type I and III IFNs [20]. The investigation assessed IFN induction by dsRNA with various motifs and observed that those containing the common motif (sense strand, 5’-C; antisense strand, 3’-GGG), including siRNAs, could induce IFN production within nanomolar ranges, and these elevated levels of IFN persisted for at least 24 to 48 h. Additionally, the present study employed RNA at a concentration of 10 µM in native gel electrophoresis and revealed that dsRNA with the common motif could facilitate the formation of RNA dimers through intramolecular G-quadruplexes (secondary structures formed by non-canonical G-G Hoogsteen base pairs), doubling the length of dsRNA. This facilitated the induction of IFNs through the binding of the 5’ end of each antisense strand to RIG-I. Notably, this type of dsRNA requires a minimum of 20 base pairs or more, and the common motif must be located at the terminal position. Any alterations, including deletion or substitution, will eliminate the immunostimulatory activity. Furthermore, methylation modification of the 5’ end of the antisense strand also abolishes immunostimulatory activity, preventing RNA from activating RIG-I [20, 44]. This new class of dual-function immunostimulatory siRNA has been proven to induce broad-spectrum immune stimulation capable of effectively inhibiting various prevalent respiratory virus infections. However, its potential in cancer treatment has yet to be explored. Therefore, it is worthwhile to investigate whether this unique immunostimulatory motif can induce the RIG-I signaling pathway in tumor cells and exert an anti-tumor immune therapeutic effect.

In this study, an immunostimulatory siRNA with specific overhanging sequence motifs was developed to target the highly expressed classical gene *BCL-2* in cancer cells. The results emphasize that our newly synthesized isi*BCL-2* demonstrates comparable silencing efficacy to traditional si*BCL-2*. However, due to the activation of the RIG-I signaling pathway, isi*BCL-2* exhibits a more pronounced effectiveness in impeding tumor cell proliferation and promoting apoptosis in both in vitro and in vivo contexts. These effects are evidenced by an increase in apoptotic cell positivity, alterations in MMP, ROS accumulation, and a significant rise in the levels of IFN-β, CXCL10, and MHC-I molecules [31–33]. Importantly, these effects are mitigated when the levels of RIG-I molecules in tumor cells are suppressed. This supports the notion that this novel class of immunostimulatory siRNA can potentiate the RIG-I signaling pathway in cancer cells, intensifying apoptotic signals and immune responses within tumor cells and tissues, thereby eliciting a stronger anti-tumor impact.

Regarding the underlying mechanism of the RIG-I/MAVS/IRF3 pathway activation by isi*BCL-2* molecules, this study, utilizing natural gel electrophoresis, revealed that isi*BCL-2* molecules containing a unique motif (sense strand, 5’-C; antisense strand, 3’-GGG) possess the ability to form dimers, thus doubling RNA length. This observation aligns with previous research findings [20], suggesting that this distinctive motif can generate highly effective RIG-I agonist dsRNA through Hoogsteen base pairing [44]. In contrast, si*BCL-2* molecules with common sequences do not exhibit this effect.

In summary, our research has demonstrated the distinctive properties of the newly synthesized isi*BCL-2*. First, compared to conventional si*BCL-2*, isi*BCL-2* with specific sequences displays an enhanced ability to induce extensive damage to tumor cells through the RIG-I signaling pathway, beyond its conventional gene silencing function. This dual functionality contributes to increased rates of tumor cell apoptosis. Second, although the immune-stimulating sequence within isi*BCL-2* has been validated in antiviral treatments previously [20], our study is the first to introduce this sequence into tumor immunotherapy. Our findings affirm that isi*BCL-2* molecules effectively stimulate the upregulation of IFN-β, CXCL10, and MHC-I levels in tumor cells *via* the RIG-I signaling pathway, promoting immune system activation and reversing the immunosuppressive tumor microenvironment. Importantly, compared to 5’-ppp siRNA, this particular class of siRNA, characterized by distinctive sequences, demonstrates superior cost-effectiveness and a simplified synthesis process [45]. Notably, previous studies have established that compared to the immune stimulant poly(I: C), this type of immune-stimulating siRNA, forming RNA duplexes with specific sequence lengths, selectively activates the RIG-I signaling pathway. This targeted activation induces a suitable immune response, avoiding excessive immune toxicity triggered by TLR or MDA5, thereby preventing tissue damage [20, 21]. Consequently, it emerges as a viable alternative for generating dual-functional siRNA.

Our study underscores the importance of careful consideration in designing siRNA sequences. Specifically, it is imperative to eliminate or modify specific immunostimulatory motifs to prevent unintended activation of innate immune responses by siRNA [16, 46]. Tailoring immunostimulatory sequences based on practical requirements in immunotherapy allows for a more adaptable and precise approach to sequence design [18, 47, 48]. The unique immunostimulatory siRNAs introduced in this study, along with their associated sequences (sense strand, 5’-C; antisense strand, 3’-GGG), present a novel therapeutic avenue and investigative perspective for siRNA applications in both viral infections and cancer immunotherapy. This research provides valuable insights into the ongoing exploration of siRNA-based therapies, emphasizing the need for strategic sequence design considerations in optimizing their therapeutic potential [42].

However, a significant challenge persists in achieving effective systemic delivery, particularly for the targeted treatment of GBM [49]. Current siRNA research focuses on chemically modifying the siRNA backbone and employing various delivery vehicles to achieve effective targeting of siRNA to glioma cells. Viral vectors, nanomaterials, dendrimers, siRNA-conjugated systems, and other extracellular vesicles have substantially attracted attention as potential delivery tools. These innovative strategies aim to enhance the stability and cellular uptake of siRNA, ultimately maximizing its therapeutic potential [49].

Our research team has successfully developed a sophisticated complex by combining epidermal growth factor receptor siRNA with cyclic Arg-Gly-Asp (cRGD) peptide, enabling specific targeting of GBM cells that overexpress *αvβ3 integrin* [50]. This approach has demonstrated a significant inhibitory effect on tumor growth in both in vitro and in vivo studies. Subsequently, the uptake of siRNA in GBM and non-small cell lung cancer was further enhanced by incorporating a bivalent cRGD peptide modification [51, 52]. Additionally, co-administration of Gelofusine helped mitigate renal damage caused by siRNA accumulation in the kidneys [53]. Our research group has recently successfully synthesized cRGD-polyethylene glycol (PEG)-siRNA molecules by covalently linking cRGD with acid-sensitive PEG compounds at the termini of both the siRNA positive and antisense strands. Experimental validation has demonstrated their efficient uptake by cancer cells within the acidic tumor microenvironment, resulting in increased targeting precision. The findings from this study will soon be published. These strategies have resulted in improved safety and precise targeting for effective treatment.

## Conclusions

In this study, we have demonstrated the potent anti-cancer properties of the newly synthesized isi*BCL-2* in GBM. Comparative analysis with si*BCL-2* reveals that isi*BCL-2* exhibits enhanced efficacy in suppressing tumors and inducing apoptosis through the activation of the RIG-I signaling pathway. This siRNA not only outperforms its counterparts but also elevates key immunomodulatory factors, including IFN-β, CXCL10, and MHC-I molecules, thereby triggering a potent immune response. Our findings underscore the potential of this novel immunostimulatory siRNA in cancer immunotherapy, providing a compelling alternative for the synthesis of dual-functional siRNA. Future research endeavors hold promise for optimizing and exploring innovative cancer treatment modalities by combining this immunostimulatory siRNA with targeted delivery systems.

## Data Availability

The data that support the findings of this study are available from the corresponding author upon reasonable request.

## Abbreviations

BCL-2: B-cell lymphoma 2
CCK-8: Cell counting kit 8
CXCL10: C-X-C motif chemokine ligand 10
Cyclic RGD: Cyclic Arg-Gly-Asp
DMEM: Dulbecco’s modified Eagle’s medium
ELISA: Enzyme-linked immunosorbent assay
FBS: Fetal bovine serum
GBM: Glioblastoma multiforme
IFN: Interferon
IHC: Immunohistochemistry
IRF3: Interferon regulatory factor 3
MAVS: Mitochondrial antiviral signaling protein
MHC-I: Major histocompatibility complex class I
MMP: Mitochondrial membrane potential
PBS: Phosphate-buffered saline
PEG: Polyethylene glycol
PI: Propidium iodide
qRT-PCR: Quantitative reverse transcription polymerase chain reaction
RIG-I: Retinoic acid-inducible gene I
ROS: Reactive oxygen species
TLR: Toll-like receptor
TUNEL: Terminal deoxynucleotidyl transferase dUTP nick end labeling
